# Oral candidal carriage and its association with dental carious lesions in asymptomatic adults: a cross-sectional study from the UAE

**DOI:** 10.1186/s12903-021-01559-3

**Published:** 2021-04-19

**Authors:** Suhail H. Al-Amad, Betul Rahman, Nadia Khalifa, Manal A. Awad

**Affiliations:** College of Dental Medicine, University of Sharjah, Room M28-130, PO Box 27272, Sharjah, UAE

**Keywords:** Candida, Dental caries, DMFS, Diabetes, Periodontal disease

## Abstract

**Background:**

*Candida’s* potential association with dental caries has previously been reported in children. This research aimed at investigating the associations between *Candida* species in whole saliva and dental and periodontal health indices in a sample of adult patients.

**Methods:**

A cross-sectional study involving 160 patients investigated the associations between *Candida* species collected by oral rinse technique, and Decayed, Missing, and Filled Surfaces (DMFS), and periodontal health indices. Chi-square and Independent Samples *t*-test were used to assess the associations between *Candida* carriage and confounding variables. Binary logistic regression was used to assess association strengths between *Candida* carriage and DMFS, adjusted for socio-demographic variables, diabetes mellitus and plaque index.

**Results:**

*Candida* colonies were identified in 49 (30.6%) patients with CFUs ranging from 10^3^ to 10^5^ colonies per mL. The quantity of *Candida* CFUs increased with age (*r* = 0.200; *p* < 0.05). Among all dental and periodontal health indices, only DMFS was significantly associated with higher values of *Candida* carriage (*p* = 0.034), and this association was independent from sex, age, smoking, diabetes mellitus and plaque index (OR: 1.014; 95% CI 1.0–1.03; *p* = 0.048).

**Conclusion:**

The association between *Candida* species in whole saliva and DMFS reported here supports an etiological role of *Candida* in dental cariogenesis among adults.

## Background

Oral candidiasis is the most common opportunistic fungal infection in the oral cavity [1]. At least 15 species of *Candida* have been identified as pathogenic to humans, with the most commonly isolated species from the oral cavity being *Candida albicans* [2]. The prevalence of oral *Candida* carriage is highest in infants and elderly [3], and ranges from 2.0 to 71.3% in adults and from 40.6 to 54.2% in infants [4].

The wide prevalence range of *Candida* carriage in adults has been attributed to a plethora of -often overlapping- local and systemic factors. Those include the presence of topographical variations in the mouth as inhabitant sites for the fungus (i.e. denture base), reduction in salivary flowrate [5], poor glycemic control in diabetic patients [6] and suppression of the immune system caused by HIV infection, chemotherapy and/or radiotherapy [7].

*Candida*’s role in causing and perpetuating dental caries has been the subject of recent debate with studies suggesting an association [8, 9] and others refuting it [10, 11]. In their meta-analysis, Xiao et al. found that children with *C. albicans* have more than five times higher odds of developing early childhood caries by comparison with those who don’t have the fungus [12].

The ability to ferment sugar and induce an acidic microenvironment that favours demineralization of dental hard tissue is a common feature of both *Candida* and the cariogenic bacteria *Streptococcus mutans* [11, 13]. Using rodent models, Falsetta et al. found that dental caries was amplified when *S. mutans* coexisted with *C. albicans*, by comparison to having one species alone [14]. It is now known that this coexistence between those two microorganisms is possible through the *S. mutans*-derived exoenzyme glucosyltransferase B (GtfB), which mediates a bond between the bacterium and the fungus allowing a stable inter-microbial microecosystem [15].

Attempts to control dental caries necessitates a good understanding of the aetiological factors that favour the growth of both cariogenic bacteria and *Candida*. Despite the wide diversity of those aetiological factors, two are considered to be among the most influential: poor oral hygiene and diabetes mellitus [16].

Most of the studies which investigated the association between *Candida* species and dental caries were reported in children, and without adjusting for plaque index and diabetes mellitus. This proposes the null hypothesis that *Candida* species has no association with dental and periodontal diseases in adults. Accordingly, the aims of this research were to report on the prevalence of *Candida* carriage among asymptomatic adults, and to investigate the associations between *Candida* species in whole saliva and dental and periodontal health indices, adjusted for plaque index and diabetes mellitus.

## Materials and methods

This study has been reviewed and approved by the University of Sharjah Research Ethics Committee (approval number: ERC/23/11/15/45) and has been performed in accordance with the ethical standards of the Declaration of Helsinki of 1964 and its later amendments. All patients who participated in this study gave their informed consent prior to their participation.

Patients attending the University Dental Hospital Sharjah for medical and dental consultations were invited to participate in a study that investigated oral health-related quality of life among diabetic patients [17]. The same sample consented to take part in a second arm which investigated the associations between *Candida* species and dental and periodontal health indices. In this paper, we are reporting the results of the latter arm. Patients who were below 18 years of age, have less than 10 natural teeth present, have been taking antibiotics and/or steroidal and /non-steroidal anti-inflammatory medications during the 3 months prior to recruitment were excluded.

Participants were asked to complete a questionnaire consisting of basic demographics and self-reported medical history. Those included sex, age, nationality, marital status, smoking, level of education, years with diabetes and the nature of diabetes treatment. A clinical examination of the oral cavity was performed by two dentists who were trained and calibrated for this research’s data collection. The clinical examination included recording the DMFS (i.e. number of dental surfaces that are decayed, missing due to caries, or filled) in accordance with the World Health Organization (WHO) standards. Dental plaque was assessed on the facial and lingual surfaces of all teeth using plaque disclosing agents and was recorded based on the Turesky’s modified Quigley–Hein plaque index. Periodontal health was assessed using Williams periodontal probe (Hu‐Friedy, Chicago, IL, USA) and recorded using the following parameters: Loss of attachment, furcation involvement, pocket depth, gingival recession, tooth mobility and bleeding on probing. All dental and periodontal indices were calculated as means and standard deviation according to the WHO standards.

*Candida* was isolated from whole saliva and quantified using the oral rinse technique [18] in which research participants were asked to gargle with 10 mL sterile phosphate buffered saline for 60 s and then to spit out the rinse into a disposable plastic container. The contents, without concentration, were sampled using 10 µL disposable inoculation loop and streaked over Sabouraud agar culture medium (Medysinal Co. Dubai, UAE) using the 13-streak method. The culture media were incubated in 37 °C for 48 h, after which the *Candida* colonies were morphologically identified and quantified by counting visible colonies and reported as Colony Forming Units (CFUs). Doubtful morphologies were further examined under the microscope using wet mount method for verification.

Statistical Package for the Social Science (SPSS) Version 26.0 (IBM Corp. Released 2019. IBM SPSS Statistics for Macintosh, Version 26.0. Armonk, NY) was used for statistical analyses. Chi-square test was used to assess associations between *Candida* as a nominal variable (positive or negative) and various demographic and medical history variables, while Independent Samples *t*-test was used to assess the associations between the presence of *Candida* and the mean scores of dental and periodontal health indices. Binary logistic regression analysis was used to assess the strength of association between *Candida* carriage and DMFS, adjusted for basic demographics, plaque index and diabetes status. Pearson’s bivariate correlation was used to assess correlations between *Candida* CFU values and the values of dental and periodontal indices. *p*-value was considered significant if ≤ 0.05.

## Results

A total of 160 patients took part in this study. The mean age of participants was 43.76 years (SD = 15.7), ranging from 18 to 80 years with equal sex distribution. Most participants were married (68%), expatriates (80%), and university graduates (61%). Almost half the sample (51%) were diabetic, the majority of whom were non-insulin-dependent (79%) (Table 1).

**Table 1:** Sample demographics and associations with oral *Candida* species carriage state

Variable	Total N (%)	*Candida*		*p *value*
		Negative N (%) 111 (69.4)	Positive N (%) 49 (30.6)	
*Sex*				
Females	80 (50)	58 (72.5)	22 (27.5)	0.391
Males	80 (50)	53 (66.3)	27 (33.8)	
*Age*				
18–30	41 (25.6)	30 (73.2)	11 (26.8)	0.403
31–50	61 (38.1)	45 (73.8)	16 (26.2)	
51–70	50 (31.3)	32 (64)	18 (36)	
71–80	8 (5)	4 (50)	4 (50)	
*Nationality*				
Expats	128 (80)	88 (68.8)	40 (31.3)	0.732
UAE nationals	32 (20)	23 (71.9)	9 (28.1)	
*Marital status*				
Single/divorced/widowed	51 (31.9)	39 (76.5)	12 (23.5)	0.183
Married	109 (68.1)	72 (66.1)	37 (33.9)	
*Smoking*				
No	123 (76.9)	85 (69.1)	38 (30.9)	0.893
Yes	37 (23.1)	26 (70.3)	11 (29.7)	
*Educational level*				
School level	63 (39.4)	42 (66.7)	21 (33.3)	0.549
University level	97 (60.6)	69 (71.1)	28 (28.9)	
*History of diabetes mellitus*				
No	78 (48.8)	57 (73.1)	21 (26.9)	0.322
Yes	82 (51.2)	54 (65.9)	28 (34.1)	
*Treatment of diabetes*				
Non-insulin dependent	64 (79)	41 (64.1)	23 (35.9)	0.335
Insulin dependent	17 (21)	13 (76.5)	4 (23.5)	

*Based on Chi-square test

*Candida* was isolated from 49 (30.6%) participants with CFUs ranging from 10^3^ to 10^5^ colonies per mL. The mean value of CFU was 3356 CFU/mL (SD = 12,102). No statistically significant difference was seen between patients with positive and negative *Candida* carriage in terms of sex, age categories, nationality, marital status, smoking, educational level, presence of diabetes mellitus and its treatment (Table 1).

Table 2 shows the associations between *Candida* positivity and various dental and periodontal health indices in which the values of DMFS, furcation involvement, pocket depth, gingival recession, and tooth mobility were higher among the positive *Candida* group. However, the only statistically significant association was between the positive *Candida* group and DMFS (*p* = 0.034). The same association was independent from sex, age, plaque index, and history of smoking and diabetes (OR: 1.014; 95% CI 1.0–1.03; *p* = 0.048) (Table 3).

**Table 2:** Differences between means of *Candida* species carriage and dental and periodontal health indices

Variable	Mean (SD)	*Candida*		*p *value*
		Negative Mean (SD)	Positive Mean (SD)	
DMFS	34.19 (27.82)	31.09 (25.15)	41.36 (32.37)	0.034
Plaque index	1.90 (1.49)	1.91 (1.70)	1.87 (0.88)	0.880
Clinical attachment loss	2.15 (1.47)	2.16 (1.43)	2.11 (1.58)	0.826
Furcation involvement	0.03 (0.10)	0.02 (0.04)	0.06 (0.18)	0.066
Pocket depth	2.00 (0.61)	1.99 (0.61)	2.05 (0.60)	0.609
Recession	0.60 (0.81)	0.56 (0.72)	0.70 (1.01)	0.385
Mobility	0.10 (0.21)	0.09 (0.19)	0.12 (0.26)	0.538
Bleeding on probing	0.55 (0.27)	0.55 (0.28)	0.54 (0.26)	0.883

*Based on Independent Samples *t *test

**Table 3:** Binary logistic regression analysis for the association between *Candida* species carriage and dental caries indices adjusted for patients’ characteristics and diabetes status

Variable	Odds ratio	95% CI		*p *value
		Lower	Upper	
Sex^†^	1.400	0.641	3.059	0.399
Age	0.996	0.969	1.024	0.797
Smoker^†^	0.802	0.326	1.971	0.630
Diabetes Mellitus^†^	1.392	0.643	3.011	0.401
Plaque index	0.897	0.658	1.222	0.490
DMFS	1.014	1.000	1.029	0.048

^†^Reference categories: Females, non-smokers, non-diabetic

Patients’ age showed a significant positive correlation with *Candida* CFU counts and the majority of dental and periodontal disease indices. *Candida* CFU counts-on the other hand-positively corrected with DMFS and gingival recession (*r* = 0.202, *p* < 0.05 for both) (Table 4).

**Table 4:** Pearson’s bivariate correlation between candida carriage colony forming units and various continuous variables

	*Candida *(*r*)	Age (*r*)	Plaque index (*r*)	DMFS (*r*)	Pocket depth (*r*)	Mobility (*r*)	Bleeding on probing (*r*)	Recession (*r*)	Furcation involvement (*r*)
Age	0.200*	–	–	–	–	–	–	–	–
Plaque index	− 0.015	0.220**	–	–	–	–	–	–	–
DMFS	0.202*	0.476**	0.162*	–	–	–	–	–	–
Pocket depth	0.106	0.396**	0.132	0.176	–	–	–	–	–
Mobility	− 0.012	0.364**	0.254**	0.243**	0.325**	–	–	–	–
Bleeding on probing	− 0.154	0.004	0.117	− 0.069	0.364**	0.220*	–	–	–
Recession	0.202*	0.595**	0.197*	0.246**	0.492**	0.571**	0.094	–	–
Furcation involvement	0.130	0.233*	0.250**	0.122	0.164	0.592**	0.186*	0.261**	–
Clinical attachment loss	0.134	0.289**	0.141	0.120	0.833**	0.568**	0.250**	0.799**	0.314**

*Significance < 0.05 level (2-tailed)

**Significance < 0.01 level (2-tailed)

## Discussion

Oral candidiasis is the most common fungal infection in humans. In its pathological form, *Candida* causes pain, altered taste sensation and dysphagia. *Candida* is a commensal microorganism that has been isolated from the oral cavity of healthy asymptomatic individuals at various rates ranging from 2 to 71% [4]. This wide range of prevalence has been attributed to alterations in local and systemic host immune defences, glycaemic control, presence of dentures and changes in salivary quality and quantity [19]. The prevalence variation has also been attributed to the different methods used by different authors to isolate and quantify *Candida* [4].

In our study, *Candida* species was isolated from 30.6% of our sample with no relationship to any of the participant’s demographics except age, whereby a significant positive correlation between the quantity of *Candida* species and the participant’s age was observed (*p* < 0.05). This finding is in agreement with many previous studies which showed higher *Candida* species counts in older -otherwise healthy-persons [4, 20].

Our sample consisted of dentate asymptomatic patients of equal sex distribution and relatively wide age range (i.e. 18–80 years). Eighty percent (80%) of our sample were expatriates living in the United Arab Emirates (UAE), which is representative of the country’s population profile whereby only 13% of the population are UAE nationals. The prevalence of *Candida* species reported here is lower than that reported in nearby Saudi Arabia by Alrayyes et al. [16] and Darwazeh et al. [21] where they found the prevalence to be 43.4% and 52%, respectively. Diversity in the prevalence of *Candida* carriage is commonly observed and has been attributed to many overlapping local and systemic factors in the studied population.

Despite its relative high prevalence among people, the potential role *Candida* species could play in causing dental and periodontal diseases, or in facilitating their progression, remains unclear and debatable. Eidt et al. reported a non-cariogenic nature of *Candida albicans* [11] and Willems et al. even suggested that *Candida albicans* has a role in preventing dental caries [22].

On the other hand, several other investigators reported an important role *Candida* plays in the process of cariogenesis [9, 13, 23]. For example, Zijnge et al., in their in-vivo study of the supragingival plaque, reported a co-habitational ecosystem of *Streptococcus* species and *Candida albicans*, which infers a relationship between *Candida* and dental caries [9]. A similar conclusion was reached by Dige and Nyvad who studied the microecology of occlusal and root caries and found that both *Candida albicans* and *Candida dubliniensis* have an integral part in the development of dental caries along with non-mutans streptococci [23]. The identification of the *Streptococcus mutans*-derived exoenzyme GtfB, which firmly mediates the bonding between the bacterium and *Candida* species supports the latter’s role in dental cariogenesis [15]. Many investigators concluded that deep dental cavities act as niches for the fungus colonization [13, 23].

In our study, we reported a significant association between DMFS scores and both the presence and quantity of *Candida* species (*p* = 0.034 and *p* < 0.05, respectively), which was independent from age, sex, smoking, diabetes and plaque index (*p* = 0.048). This association is in agreement with previous studies that reported similar findings [8, 9]. Whilst most of those studies were assessing the relationship between *Candida* species and dental caries in children, our study presents an evidence of an association between the fungus and dental caries in an adult population. This association was adjusted for the most common factors that have been attributed to both oral candidiasis and dental caries. Among those factors are poor oral hygiene and diabetes mellitus.

Higher rate of carriage of *Candida* species has been associated with diabetes mellitus, and more so in patients with poor glycaemic control [24]. The prevalence of diabetes mellitus in the UAE is among the highest in the world, hence it was necessary for this confounding factor to be adjusted for in our sample. Despite the high prevalence of diabetes in the UAE, and the relatively high percentage of diabetic patients recruited in our study (i.e. 51%), diabetes status was not associated with *Candida* carriage status (*p* = 0.322), nor has it shown an effect on the association between *Candida* species and DMFS (*p* = 0.401).

*Candida* carriage has also been associated with poor oral hygiene [25], which is also highly prevalent in the UAE, particularly among children [26]. The mean plaque index (as an objective marker of oral hygiene) in our sample was 1.9 (SD = 1.49) with no difference between positive and negative *Candida* carrier state (*p* = 0.880). Moreover, this variable had no effect on the association between *Candida* species and DMFS (*p* = 0.490).

The prevalence of severe periodontal disease in the Middle East has been estimated to be 10.4%, which is less than the age-standardized global prevalence of 11.2% [27]. Periodontal diseases have shown strong associations with diabetes mellitus [28] and tobacco smoking [29], with a recent emphasis on the role *Candida* plays in the development and progression of periodontal disease [30].

Although periodontal health indices showed positive correlations among themselves, they were not associated with the presence nor the quantity of *Candida* in our study. This could be attributed to the sampling method used here which was based on whole saliva rather than direct sampling from periodontal pockets.

Despite our findings, we observe a number of limitations related to the methodology used in this study. Firstly, the collection method used was based on the oral rinse technique, which although has the advantage of sampling the entire oral cavity’s macro-environment, it does not show the direct relationship between the fungus and the dental carious lesions. Secondly, the identification of *Candida* species was solely based on their colony morphology without the use of molecular biology to identify its strains. The outcomes of this study should -therefore- be limited to the generic presence or absence of *Candida* species in the oral cavity. Thirdly, hyposalivation is a common predisposing factor that has been associated with both dental caries and oral candidiasis [31], which was not assessed in this study. Finally, although the most common predisposing factors for both oral candidiasis and dental caries were adjusted for, other predisposing factors, such as the presence of partial denture and frequency and duration tobacco smoking, weren’t assessed in this study.

The association between *Candida* species in whole saliva and DMFS, that was independent from sex, age, smoking, plaque index and diabetes mellitus, is a finding that strengthens the assumption that *Candida* species might not simply be normal oral flora with the capacity of causing opportunistic fungal infections, but a microorganism with an active role in dental cariogenesis. The value of medically controlling oral yeast by antifungal medications, and the effect this would have on dental caries control, remains to be investigated in future clinical trials.

## Data Availability

Raw anonymous data is available on the Corresponding Author’s Google drive privileged account. A link to this data can be provided upon reasonable request.
